# The Contrasting Effects of an Action Video Game on Visuo-Spatial Processing and Proactive Cognitive Control

**DOI:** 10.3390/ijerph17145160

**Published:** 2020-07-17

**Authors:** Robert West, Edward L. Swing, Craig A. Anderson, Sara Prot

**Affiliations:** 1 Department of Psychology and Neuroscience, DePauw University, Greencastle, IN 46135, USA; 2 Phoenix Children’s Hospital, Phoenix, AZ 85016, USA; eswing83@gmail.com; 3 Department of Psychology, Iowa State University, Ames, IA 50010, USA; caa@iastate.edu; 4 School of Psychological, Social, and Behavioral Sciences, Coventry University, Coventry CV1 5FB, UK; sara.prot@coventry.ac.uk

**Keywords:** action video games, proactive cognitive control, visuo-spatial processing, attention

## Abstract

First person shooter or action video games represent one of the most popular genres within the gaming industry. Studies reveal that action gaming experience leads to enhancements of visuo-spatial processing. In contrast, some correlational evidence reveals that experience with action video games may be associated with reduced proactive cognitive control. The two primary goals of the current study were to test the causal nature of the effect of action gaming on proactive cognitive control and to examine whether an increase in visuo-spatial processing and a decrease in proactive cognitive control arise from the same amount of experience playing an action video game. Participants completed tasks measuring visuo-spatial processing and cognitive control before and after 10 practice sessions involving one of three video games or were assigned to a no gaming experience control group. The data revealed the typical increase in visuo-spatial processing and a decrease in proactive, but not reactive, cognitive control following action game training. The sizes of these two training effects were similar in magnitude, but interpretation of the effects was constrained by baseline differences between the four groups of subjects. The possibility of a causal effect of action gaming on proactive cognitive control is interesting within the context of correlational evidence linking greater action gaming experience to reduced cognitive control, poor decision making, and increased impulsivity.

## 1. Introduction

Playing video games represents a ubiquitous form of entertainment beginning in childhood and continuing into adulthood. According to the Entertainment Software Association [1], one of the most popular genres of video games is first person shooter games that have also been labeled action [2] or violent [3] video games in the scientific literature. In these games, a player navigates through a virtual environment with the goal of terminating threats or enemies, or destroying enemies’ resources. Some popular titles in this genre include Doom, Halo, Call of Duty, and Gears of War, among others. Meta-analytic work demonstrates that experience with video games in general, and action games in particular, has reliable effects on perception and cognition [4,5,6,7], as well as, social information processing and social behavior [3]. In the cognitive literature the effects of action games have typically been characterized as positive or beneficial (e.g., an expansion of the useful field of view or an increase in the ability to track multiple objects moving in space [4]), whereas in the social psychological literature the effects of these games often reflect negative or undesirable outcomes (e.g., increased aggression, decreased prosocial behavior, blunted emotional responses) [3].

Extended exposure to action video games is associated with improvements in visuo-spatial, visuo-motor, and auditory information processing [4,8,9]. This improvement is seen in both low level processes underpinning visual acuity and contrast sensitivity [10] and higher level attentional processes that facilitate the detection of targets among distractors [2] and the tracking of multiple objects through space over time [11]. Several studies demonstrate that a durable effect of action gaming can arise from as little as 10 h of gaming experience or training [2,12]. Furthermore, this experience may serve to reduce the impact of gender differences in spatial processing and last for several months [12]. These findings have led to the suggestion that action games could serve as a platform for the rehabilitation of perceptual, attentional, and motor deficits [4,9].

Meta-analytic work has established the beneficial effect of action gaming on visuo-spatial processing across a number of different tasks and research groups [5], although the effect is not observed universally [13]. Green and others have consistently observed that action gaming is related to improvements of performance in the useful field of view (UFOV) task in studies using both individual difference and training designs [2,4,12]. The effect of action gaming on performance of this task is interesting within the context of a program of research that utilizes a standardized version of the UFOV task. This research demonstrates that training related enhancement of the UFOV in older adults has a number of positive outcomes. These include a delay in driving cessation [14], improved activities of daily living [15], and even a reduced risk of Alzheimer’s disease [16]. Given the consistency of the effect of action gaming on performance of the UFOV task, and the practical benefits of improving perceptual and cognitive processing related to the UFOV, a version of the task modeled after the work of Greene and Bavelier [2] served as the index of visuo-spatial processing for the current study.

In contrast to the beneficial effects of action gaming on visuo-spatial processing, other evidence reveals that exposure to digital entertainment media, including video games, may be associated with a decrease in impulse control and an increase in pathological attention related to attention deficit hyperactivity disorder (ADHD) [17,18]. For instance, the findings of a meta-analysis reveal that there is a reliable relationship between screen media use and clinical deficits of attention and impulsivity [19]. Furthermore, other evidence indicates that the association between overall and violent media exposure and pathological attention may partially account for the effect of media exposure on aggression [20].

Consistent with findings linking media exposure and pathological attention [20], some evidence reveals that action gaming in particular may be associated with a reduction of proactive cognitive control [21], in addition to increased impulsivity and risk taking, and poor decision- making [22]. Within the dual mechanisms of control (DMC) account [23], proactive control is associated with the active maintenance of goal-relevant information that serves to bias systems supporting attention, perception, and action to achieve goal-directed behavior. In contrast, reactive control represents the just-in-time allocation of processes supporting attention and selection when interference is detected. These two forms of control represent both reliable and dissociable constructs [24]; and the use of proactive and reactive control can vary with situational factors related to task structure [25], or individual [26] or group differences [27].

Bailey et al. [22] examined the association between action gaming and proactive control by varying the response-to-stimuli interval (RSI) in the Stroop task. When the task placed minimal demands on proactive control (i.e., the RSI was 500 ms) the conflict adaptation effect—an index of the tuning of cognitive control over trials—was similar in action gamers and non-gamers. In contrast, this effect was significantly smaller in action gamers relative to non-gamers when the task placed high demands on proactive control (i.e., the RSI was 2000 ms). Converging with these behavioral data, sustained event-related brain potentials (ERPs) related to conflict adaptation were initially similar in amplitude for action gamers and non-gamers; however, this ERP activity was attenuated in action gamers later in the epoch. These data led to the conclusion that action gamers could modulate control based upon changing task demands, but were then unable to proactively maintain control for as little as two seconds between trials [22]. Consistent with these findings, other researchers have observed a negative correlation between performance on the Stroop task and action gaming when the task would likely promote proactive control [28]. Additionally, other work reveals the under-recruitment of the cognitive control network involving the anterior cingulate and lateral frontal cortex by high gamers during performance of the Stroop task [29] and while playing an action video game [30].

Motivated by evidence revealing the possibility of differential effects of action gaming on proactive cognitive control and visuo-spatial processing, the current experiment had two major goals. First, we sought to determine whether action gaming has a causal effect on proactive cognitive control, as the extant evidence is correlational. Second, we examined whether an increase in visuo-spatial processing and decrease in proactive control resulting from action gaming arise in parallel from the same amount of gaming experience. To this end, participants performed a Stroop task that is sensitive to variation in proactive and reactive cognitive control [23,25] and a UFOV task that is sensitive to the effect of action game training on visuo-spatial processing [2] in two sessions (i.e., pre-training and post-training). Between the pre- and post-training sessions, participants either played an action game (i.e., Unreal Tournament) or one of two non-action games (i.e., The Sims or Faster Than Light (FTL)) for ten 50–55 min sessions spread over 3–10 weeks, or were assigned to a no game control group. The last video game training session and the post-training session were conducted on different days. Given the extant literature, we predicted that action game training would enhance visuo-spatial processing on the UFOV task and decrease proactive control on the Stroop task. Furthermore, these effects should be observed in both absolute terms, and relative to the three control conditions that were not expected to differ from one another. We also expected there to be no effect of gaming on reactive control [21], other that there could be a general practice effect from the pre- to post-training sessions.

## 2. Materials and Methods

### 2.1. Ethics Statement

The Institutional Review Board of Iowa State University approved the study (IRB#: 11-047). Signed informed consent was obtained from research participants at the beginning of the pre-training session. The consent form described the nature of the study, the duration of training, and compensation for the study.

### 2.2. Participants

Seventy-seven participants (44 males, 32 females, 1 not reported) recruited from the Department of Psychology participant pool met the following requirements for inclusion in the final sample: (a) reported that they typically played video games fewer than 5 h per week; (b) completed the pre- and post-training measures at least three weeks and no more than 12 weeks apart; and (c) had complete data on the Stroop and UFOV tasks (No game n = 24, FTL n = 16, Sims n = 19, Unreal Tournament n = 18). Table 1 includes frequencies regarding the status of 175 individuals that were recruited for the study. More participants were lost from the gaming groups than the no gaming groups because individuals did not need credit beyond the pretest. This could be expected based upon differences in the amount of credit associated with the training conditions versus the No Game condition. More participants were lost from the no-game group for completing the study in less than 21 days. Age ranged from 18–24 (M = 19.57). Individuals received course credit for participation.

### 2.3. Video Games

The action video game was Unreal Tournament 2004 (UT), a fast-paced first-person shooter game. For the most part, players must continuously scan for enemies and shoot anything that moves. This action game was selected because it has been used in prior training studies that have yielded significant improvements in visuo-spatial processing [2], and because game-play requires quick reactions to changes in the visual field. The two non-action video games were The Sims 2 (a social simulation video game used as a control game in some previous studies [10] and Faster Than Light (FTL, a real-time strategy game). The Sims 2 has been used as a control game in some previous research and appears to be engaging over time, leading to better persistence of participants over training sessions, than a game like Tetris that is sometimes used as a control game. FTL was chosen as it has some action that is similar to Unreal Tournament and does have some aggression related to destroying enemy ships. However, FTL does not include the first person perspective or violence that is manifest in Unreal Tournament; more importantly, it does not have the same fast reaction pace of UT. The no-game participants came to the lab for only the pre- and post-training sessions.

### 2.4. Measures

Stroop task. Four color words (red, yellow, blue, green) were presented in congruent (e.g., RED presented in red, four stimuli) and incongruent (e.g., RED presented in blue, 12 stimuli) trials. The task included three phases: key mapping, practice, and test. In the key mapping phase individuals learned the color-to-key associations (color/key associations). This phase included 40 trials (i.e., 10 for each color) where a string of 10 Xs was presented until a response was made. In the practice phase, participants completed 24 (12 congruent and 12 incongruent) trials to become familiar with the Stroop stimuli. In the test phase, individuals completed two blocks of 48 trials. The mostly congruent block included 36 congruent stimuli and 12 incongruent stimuli, whereas the mostly incongruent block included 12 congruent stimuli and 36 incongruent stimuli. The order of the mostly congruent and mostly incongruent blocks was counterbalanced between participants. For all trials there was a 1000 ms inter-stimulus interval between the response and onset of the next stimulus.

Measures of proactive and reactive control were derived from performance of the test phase of the Stroop task, and were based upon response time for correct trials. Reactive control was defined as the typical Stroop effect and represented the difference in mean response time between incongruent and congruent stimuli collapsed across the two proportion-of-congruent-trials blocks. To be consistent with the other two outcome measures, we rescaled reactive control so that higher scores from the pretest to the posttest would represent better performance (i.e., less interference) by multiply the reactive control scores by −1. Proactive control was defined as the effect of the proportion of congruent trials manipulation on response time, computed as: (mostly congruent incongruent response time—mostly incongruent incongruent response time) + (mostly incongruent congruent response time—mostly congruent congruent response time). For proactive control higher scores represent a stronger effect of task context (i.e., the proportion congruent manipulation). The logic underlying the calculation of the proactive control scores is similar to that used when calculating the conflict adaption effect related to trial-to-trial modulation of proactive control. This measure of proactive cognitive control was adopted in favor of the conflict adaption effect that can be relatively small in size and can also be confounded with stimulus and response repetition or priming effects. Our measure is conceptually similar to the measure of proactive control described by Gonthier et al. [24] in their Figure 1 wherein the proportion of congruent trials between blocks modulates response time for both congruent and incongruent trials.

**Figure 1:**
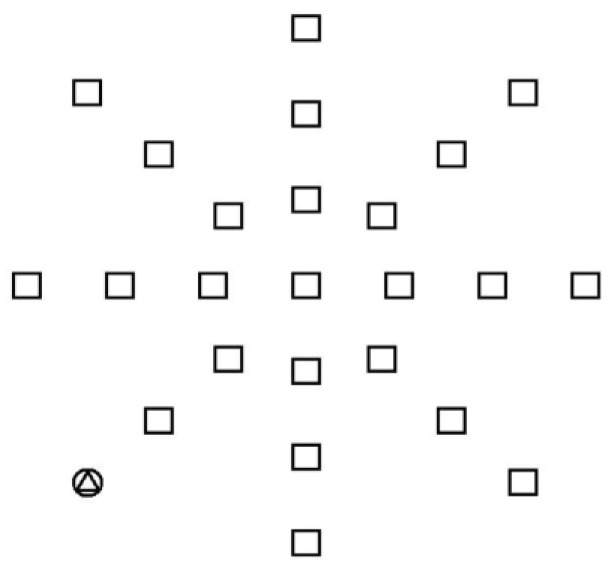
Sample target display for the useful field of view task. The target presented at 30 degrees in the southwest direction.

Useful field of view task, the task was modeled after that used by Green and Bavelier [2]. For each trial, 24 stimuli (23 distractors and one target) were presented on the eight spokes of a virtual radial array at 10°, 20°, and 30° from the center of the display. The target was a triangle embedded in a circle; the distractors were unfilled squares (Figure 1). For each trial, a fixation cross appeared in the center of the screen. Then stimuli were presented for 17 ms, followed by a 500 ms visual mask, and then a fixation cross until a response. Individuals pressed one of eight keys to indicate the spoke on which the target appeared. The response-to-stimulus interval was 1000 ms. Participants were instructed to give their best guess when they were unsure of the target’s location. The task included 24 practice trials and 96 test trials. The target appeared at each of the 24 locations equally often. The performance measure represented the percentage of correct choices collapses across distance and spoke.

### 2.5. Procedure

Only students who usually played video games less than 5 h per week were invited to participate. At the pre-training session participants were asked to confirm that they typically played video games fewer than 5 h a week, completed the Stroop task, the UFOV task, demographic items, and several individual difference measures. Participants were randomly assigned to one of the video game conditions (Unreal Tournament, Sims 2, Faster than Light) or to the no-game condition. Participants in the video game conditions signed up for 10 sessions (about 9 h) of video game play. These sessions consisted entirely of playing the assigned video game. Sessions were scheduled so that participants completed no more than 2 h of video game play on a given day. The average number of days between the pre- and post-training sessions did not significantly differ across the four groups (no game M = 31.67, SD = 12.95; FTL M = 31.80, SD = 7.63; Sims M = 33.90, SD = 9.18; Unreal Tournament M = 38.56, SD = 14.75), F(3, 72) = 1.40, *p* = 0.25.

### 2.6. Power Analysis

A power analysis was not performed at the outset of the study; at that time our primary consideration for estimating relevant sample size was based upon the extant literature examining the effect of action games on visuospatial processing in studies by the Rochester group [5]. Training studies from this laboratory tend to include 10–20 subjects per group, so our initial target was ≥20 participants per group. This target was realized in our full sample; however, loss due to attrition during the training and missing data pushed the final group numbers below this target. One can obtain a reasonable estimate of a priori power for the design based upon the published meta-analytic effect size [5]. This paper reports an average effect size for visuo-spatial processing in studies published by the Rochester group of d = 0.54. Given this value, 88 participants would be required to obtain a power of 0.80, which is close to the size of our final sample. Given the effect size for the contrasts of the Unreal Tournament group with the average of the other three groups in our data, the achieved power for proactive control was 0.76 and for UFOV it was 0.74, assuming the unbalanced design. Estimating a priori power using the observed effect sizes indicates that 88 or 92 participants would be required to obtain power of 0.80 for proactive control and UFOV, respectively. Given these values, we believe that our design is reasonably powered, and reiterate that the predicted effects for UFOV and proactive control were observed and of similar magnitude.

## 3. Results

As described in the Methods and Materials, we calculated effect scores for each of three dependent variables (i.e., proactive control, reactive control, and UFOV) for the pre- and post-training sessions. These effect scores were then transformed to a common scale by dividing each by the sample standard deviation across pre-training and post-training for the analyses of the training effects. This is statistically equivalent to creating z-scores, except that the mean is not set to zero. Positive scores indicate improved performance post-training. The data are available at osf.io/f9dbv.

First we examined whether the three control groups differed on any of the dependent measures using the error-term for the full sample. Using the error-term from the three control groups did not change the results in any substantial way. The three groups did not differ, *F*s(2, 71) < 2.07, *p*s > 0.13. These results support the planned contrasts that compare the UT group to the other three groups presented in the analyses that follow.

We then conducted a 4 (gaming group) × 3 (dependent measure: UFOV, proactive control, reactive control) × 2 (session: pretest, posttest) repeated measures ANCOVA with days between the pre- and post-training sessions as the covariate. As a reminder, we predicted that the Unreal Tournament group would improve more on the UFOV task than the other groups, and that this group would demonstrate a reduction in proactive cognitive control, relative to the three control groups. Planned contrasts were used to test these predictions. The effect of the covariate was not significant in any of the analyses, *Fs* < 1.00.

**Figure 2:**
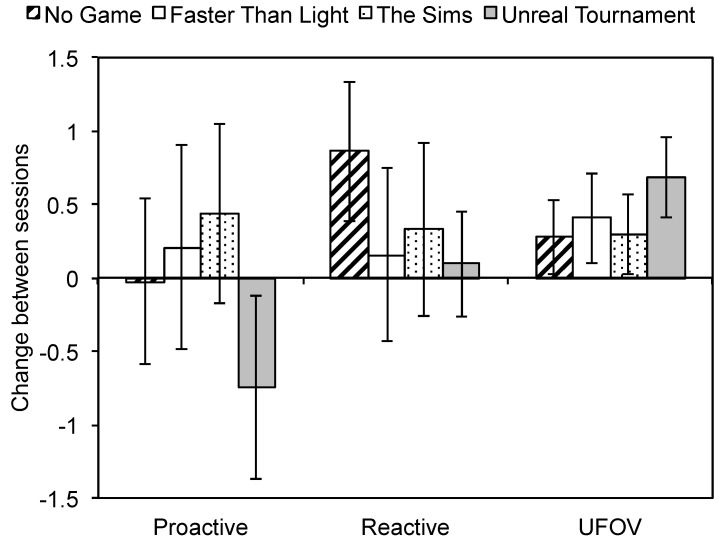
Effects of game training on visuo-spatial processing, proactive, and reactive control. Proactive: proactive control, Reactive: reactive control, UFOV: useful field of view. The error bars represent the 95% confidence interval.

The mean deviation scores for the pre- and post-training sessions are presented in Table 2. The mean and standard deviations for mean response time (Table A1) and accuracy (Table A2) in the Stroop task and accuracy in the UFOV task (Table A3) are reported in Appendix A. As predicted, the three-way interaction of gaming group × dependent measure × session was significant, *F*(6, 142) = 2.43, *p* = 0.029, in the full ANCOVA, and for the contrast comparing the Unreal Tournament group to the other three groups, *F*(2, 142) = 4.50, *p* < 0.013. As shown in Figure 2, the interaction resulted primarily from an increase in UFOV performance and a decrease in proactive control in the Unreal Tournament group, relative to the other three groups. Planned comparisons confirmed our specific predictions, as the two measures expected to be affected by action game training yielded significant contrasts: proactive control, *F*(1, 71) = 6.61, *p* = 0.012, *d* = 0.68, and UFOV, *F*(1, 71) = 5.36, *p* = 0.023, *d* = 0.61. Additionally, for the Unreal Tournament group the decrease in proactive control, *t*(18) = −2.23, *p* = 0.039, *d* = 0.66, and increase in UFOV, *t*(18) = 4.50, *p* < 0.001, *d* = 0.75, was significant in absolute terms as well. Consistent with the findings of Bailey et al. [21], the contrast for reactive control was not significant, *F*(1, 71) = 1.70, *p* = 0.196, *d* = 0.39.

These findings support the hypothesis that action video game experience may have contrasting effects on proactive cognitive control and visuo-spatial processing, although conclusions related to the causal nature of the effect are limited by the baseline differences in proactive control and visuo-spatial processing observed between the four groups. Interestingly, in the current data, the size of the effects of action gaming experience—as measured by Cohen’s d—was similar for proactive cognitive control and visuo-spatial processing.

## 4. Discussion

The current study replicated the well-documented finding that action video game training in young adults who are relatively naïve gamers enhances visuo-spatial processing in the UFOV task [2,4]. Importantly, the size of this training effect was within the confidence interval for visuo-spatial processing reported in the meta-analysis of Powers et al. [5], which included a variety of visuo-spatial tasks. This finding leads to the suggestion that our study captured similar processes as those contributing to training effects in the extant literature. The effect of action game training on visuo-spatial processing is especially important as it sets the stage for the more important contribution of the current study, the simultaneous testing of the effects of action gaming on visuo-spatial processing and proactive cognitive control. Our data demonstrate that a relatively limited amount of action gaming (i.e., about 9 h) can lead to a reduction in proactive cognitive control in the Stroop task. The effects of action gaming seen in the Stroop task are consistent with earlier correlational findings using a different index of proactive cognitive control [21]; revealing both the generality and potential causal nature of the effect of action gaming on this aspect of cognitive or executive control.

Documenting the causal effect of action gaming on proactive control is important for at least three reasons. First, these results are important given the association between media use and attention/impulsivity problems more generally [17,18], and action games and impulsivity more specifically [22]. Our findings suggest that the link between action gaming and impulsivity may not simply represent a spurious association that arises from intrinsic individual differences between action gamers and non-gamers. Instead, the current data lead to the suggestion that action gaming may represent a genuine risk factor for the reduction of cognitive control that could result in the development of impulsivity and possibly pathological attention. Based upon our preliminary findings and the results of prior research [20,22], it seems that additional research is warranted to further explore the relationship between action gaming, cognitive control, impulsivity, and pathological attention.

More broadly, there is a growing literature suggesting that screen time is associated with attention and impulsivity problems [31,32], but we know of only one experimental training study reporting such an effect. That study, presented as a conference paper [33], used functional magnetic resonance imaging to assess changes in fronto-parietal brain activity related to cognition. Thus, the demonstration of a possible causal effect of action gaming on proactive cognitive control may reveal one of the pathways by which this media exposure could contribute to the development of attention problems related to impulsivity. However, the baseline difference in proactive cognitive control and UFOV between the four groups (i.e., elevated proactive control and reduced visuo-spatial processing in the Unreal Tournament group) represents a significant confound that limits our ability to draw strong causal inferences about the effect of action gaming in the current dataset. Given this, it is clear that further research is required to further establish the robustness of the effects of action gaming on proactive control and visuo-spatial processing observed in our dataset.

Second, many news stories, the video and brain gaming industry, gamer sites, and some scholars [4,5] may have over-generalized the legitimate finding—that action games can improve visuo-spatial processing—to support an illegitimate claim—that action games are “good for improving attention.” Indeed, some have recommended that parents buy their children such fast-paced action/violent games to either ward off real-world attention problems, such as attention deficit disorder, or to aid children who already have this or related disorders. The present study provides some evidence demonstrating that the effects of action gaming on visuo-spatial processing and proactive cognitive control (i.e., one aspect of executive function) are not the same, and may in fact be opposite. What is less clear in the research literature is the extent to which different types of executive function underlie problems with real world attention and impulsivity; a question that should be explored in future research.

Third, the observed difference in the effect of action gaming on proactive and reactive cognitive control is interesting within the context of some individual-differences work. For example, Unsworth et al. [34] reported that the Stroop effect was not significantly correlated with video gaming experience in a task that would likely encourage a reactive mode of processing (i.e., stimuli were mostly congruent). Our data converge with this finding, and extend it to a training study. Our results are also interesting within the context of meta-analytic work demonstrating variability in the effect of video games on different aspects of executive function [5,7]. Here we demonstrate that some of this variability may be related to the specific aspects of cognitive control or executive function that are being measured, even within a single task (i.e., the differential effects on proactive and reactive cognitive control).

Green et al. [8] provides compelling evidence that the effect of action gaming on visuo-spatial processing results from enhancements of information integration that extend to the visual and auditory domains. Using a neural network model, these researchers demonstrated that an increase in the strength of feed forward connectivity between layers of the network was sufficient to capture differences between action gamers and non-gamers in tasks measuring the discrimination of visual motion direction and auditory tone location. Additionally, changes in this model parameter also captured the effect of action video game training in these tasks. The potential effect of action gaming on information integration is consistent with evidence from studies using ERPs to examine the neural basis of game based training effects on visual processing in older adults. These studies have revealed training related changes in ERPs that are related to increased efficiency in early perceptual and attention processing in addition to stimulus categorization [35,36]. There are some differences in the UFOV task used as an outcome in the video game training literature and as an intervention in the aging literature [14], so one avenue for future research could be to seek to establish a direct link from action game training to improved real world outcomes related to attention and cognition.

In contrast to the basis for the effect of action gaming on visuo-spatial processing, the locus of the effect of action gaming on proactive control is unclear at this time. One possibility is that the need to be receptive to rapidly changing conditions within the game in order to successfully deal with the rapid emergence of threats may bias action gamers to a reactive mode of cognitive control, and that this then translates to ventures beyond the gaming environment. The findings of the current training study, in combination with correlational evidence that link action gaming to reduced proactive control [21] further suggest that whatever the specific locus, the deleterious effects persist well after the gaming device is turned off.

There were some unexpected findings in the data that may warrant further consideration in future research. First, the high level of proactive control in the Unreal Tournament group for the pre-training session is curious. This does not appear to result from an outlier in the pre-training session. Given this, one possibility is that the high level of proactive control exhibited in the pre-training by the Unreal Tournament group reflects a limitation of the random assignment procedure. As mentioned above, this aspect of the data indicate that replicating the training effect of action gaming on proactive cognitive control should be pursued in future research. Second, while the change in reactive control from pre-training to the post-training was not significant when all four groups were included in the analysis, reactive control did increase in the no-game group across sessions (i.e., the confidence interval for the change does not include zero). It is possible that there was a general effect of gaming that served to attenuate the practice effect on reactive control relative to the no-game group, although it is unclear what the locus of this effect might be as the three games are likely associated with very different cognitive processes [5]. Additionally, the effect of session in the no-game group seems to be a rather large practice effect given the limited number of trials in the task and the fact that the session are separated by more than one month.

## 5. Conclusions and Future Directions

We have demonstrated that the commonly observed increase in visuo-spatial processing arising from playing action video games [5,11] can be accompanied by a decrease in proactive cognitive control. These contrasting effects were of similar magnitude, and arose with only nine hours of gaming experience. The current findings provide a second within-person demonstration of the simultaneous beneficial and detrimental effects of action gaming training that compliment previous work from our research group demonstrating enhancement of visual processing and attenuation of affective information processing after 10 h of action, but not non-action, game training [37]. The possible reduction in proactive cognitive control, along with the negative effects of action gaming on social and affective information processing [3,38], may serve to temper the enthusiasm expressed by some scholars regarding the potential utility of this media as a foundation for interventions designed to enhance or rehabilitate visual processing [4,9]. Furthermore, this evidence leads us to suggest that it may be worth weighing the potentially positive and negative effects of action games when considering the extension of this medium to therapeutic contexts.

After the data for the current study were collected, Gonthier et al. [24] provided a detailed analysis of task conditions in a picture-word Stroop task that allowed the researchers to dissociate proactive and reactive cognitive control, and also demonstrate that these two means of control represented distinct constructs. The approach taken by Gonthier et al. [24] used a combination of block wise and item specific proportion congruent effects that is beyond the scope of the design of the simple color-word proportion congruent Stroop task used in our study. Given the promising findings of Gonthier et al. [24], it may be worth exploring the application of this approach in further research to examine the relationship between action gaming, and other forms of video games, and different control processes.

The current study used games that are fairly old in terms of their release date. The choice of games was due to an interest in replicating (i.e., for visuo-spatial processing) and extending (i.e., for proactive cognitive control) the findings of previous research. In the context of the games used for training in the study, two of the reviewers wondered how the current findings might be influenced by recent developments in video games including the move toward more immersive virtual reality and multiplayer games in the action gaming series that add an element of cooperation and empathetic elements. It seems that more immersive game play could serve to enhance the effects of gaming on visuo-spatial processing. In contrast, the possible effects on proactive control are less clear. For instance, the need for coordination among players could serve to strengthen proactive cognitive control and enhance empathetic concern. However, it is also possible that cooperation within games could also have the negative consequence of strengthening in-group versus out-group biases depending upon the representation of individuals or groups within the game environment [39].

## Figures and Tables

**Table 1:** Frequencies for distribution of participants in the four groups for those that had complete data and those that were not included in the analyses for various reasons.

	No Game	FTL	Sims	UT	Total
Complete	24	16	19	18	77
Credit	0	9	7	11	27
Dropped Out	2	9	5	10	24
Missing	8	2	3	4	17
Not Assigned	2	1	1	1	7
Time	9	0	3	0	12
Video Game	6	2	0	2	10
Total	51	39	38	46	174

Note: Complete: complete data at pre and post test, Credit: dismissed after pretest as did not need additional course credit, Dropped Out: withdrew from the study before completing training, Missing: missing data at pre or posttest, Not Assigned: signed up for appointment but did not attend pretest, Time: less than 21 days between pre and posttest, Video Game: reported playing more than 5 h a week of video games in pretest. FTL = Faster Than Light, UT = Unreal Tournament.

**Table 2:** Mean deviation scores and standard errors for proactive control, reactive control, and UFOV for the pre-training and post-training adjusted for the number of days between the two testing sessions.

	Proactive Pre	Post	Reactive Pre	Post	UFOV Pre	Post
No Game	0.03 (0.19)	0.003 (0.21)	−1.78 (0.18)	−0.92 (0.23)	1.47 (0.20)	1.74 (22)
FTL	0.13 (0.24)	0.34 (0.27)	−1.13 (0.22)	−0.97 (0.29)	1.55 (0.24)	1.96 (0.27)
Sims	0.12 (0.21)	0.56 (0.24)	−1.42 (0.20)	−1.09 (0.25)	1.69 (0.22)	1.98 (0.24)
UT	1.18 (0.21)	0.44 (0.24)	−1.59 (0.20)	−1.50 (0.26)	1.25 (0.22)	1.94 (0.24)

Note: UFOV = Useful field of view, UT = Unreal Tournament, FTL = Faster Than Light.

## Appendix A

**Table A1:** Mean and standard deviation for response time in Stroop task at the pretest and posttest as a function of congruency and proportion of congruent trials and group.

		No Game	FTL	Sims	UT
Pretest	MC Cong.	689(127)	689(110)	720(114)	793(196)
	MC Inc.	855(182)	827(124)	879(150)	1061(299)
	MI Cong.	691(161)	692(137)	724(120)	874(215)
	MI Inc.	843(178)	798(135)	860(136)	972(248)
Posttest	MC Cong.	681(119)	655(104)	646(88)	735(186)
	MC Inc.	788(131)	792(183)	811(117)	923(235)
	MI Cong.	721(152)	666(108)	709(138)	779(216)
	MI Inc.	770(125)	737(143)	776(117)	899(235)

Note: FTL = Faster Than Light, UT = Unreal Tournament, MC = mostly congruent, MI = mostly incongruent, Cong. = congruent, Inc. = incongruent.

**Table A2:** Mean and standard deviation for response proportion correct in Stroop task at the pretest and posttest as a function of congruency and proportion of congruent trials and group.

		No Game	FTL	Sims	UT
Pretest	MC Cong.	0.97(.04)	0.99(0.02)	0.99(0.02)	0.98(0.029)
	MC Inc.	0.93(0.093)	0.93(0.085)	0.94(0.077)	0.92(0.077)
	MI Cong.	0.97(0.046)	0.98(0.046)	0.97(0.05)	0.95(0.095)
	MI Inc.	0.94(0.063)	0.95(0.047)	0.94(0.062)	0.94(0.054)
Posttest	MC Cong.	0.98(0.026)	0.96(0.062)	0.98(0.017)	0.98(0.025)
	MC Inc.	0.95(0.084)	0.88(.133)	0.95(0.075)	0.93(0.100)
	MI Cong.	0.97(0.054)	0.96(0.052)	0.99(0.025)	0.98(0.048)
	MI Inc.	0.93(0.050)	0.93(0.080)	0.95(0.051)	0.95(0.053)

Note: FTL = Faster Than Light, UT = Unreal Tournament, MC = mostly congruent, MI = mostly incongruent, Cong. = congruent, Inc. = incongruent.

**Table A3:** Mean and standard deviation for proportion correct in the UFOV task at the pretest and posttest as a function of group.

	No Game	FTL	Sims	UT
Pretest	0.48(0.261)	0.45(0.314)	0.51(0.302)	0.38(0.267)
Posttest	0.57(0.298)	0.56(0.336)	0.60(0.324)	0.60(0.278)

Note: FTL = Faster Than Light, UT = Unreal Tournament.
